# Stroke in SARS-CoV-2 Infection: A Pictorial Overview of the Pathoetiology

**DOI:** 10.3389/fcvm.2021.649922

**Published:** 2021-03-29

**Authors:** Saeideh Aghayari Sheikh Neshin, Shima Shahjouei, Eric Koza, Isabel Friedenberg, Faezeh Khodadadi, Mirna Sabra, Firas Kobeissy, Saeed Ansari, Georgios Tsivgoulis, Jiang Li, Vida Abedi, Donna M. Wolk, Ramin Zand

**Affiliations:** ^1^Neuroscience Research Center, Guilan University of Medical Sciences, Rasht, Iran; ^2^Neurology Department, Neuroscience Institute, Geisinger Health System, Danville, PA, United States; ^3^Geisinger Commonwealth School of Medicine, Scranton, PA, United States; ^4^Department of Biology, Pennsylvania State University, State College, PA, United States; ^5^PES University, Bengaluru, India; ^6^Neurosciences Research Center (NRC), Lebanese University/Medical School, Beirut, Lebanon; ^7^Program of Neurotrauma, Neuroproteomics and Biomarker Research (NNBR), University of Florida, Gainesville, FL, United States; ^8^National Institute of Neurological Disorders and Stroke, National Institute of Health, Bethesda, MD, United States; ^9^Second Department of Neurology, School of Medicine, “Attikon” University Hospital, National and Kapodistrian University of Athens, Athens, Greece; ^10^Department of Molecular and Functional Genomics, Geisinger Health System, Danville, PA, United States; ^11^Biocomplexity Institute, Virginia Tech, Blacksburg, VA, United States; ^12^Molecular and Microbial Diagnostics and Development, Diagnostic Medicine Institute, Laboratory Medicine, Geisinger Health System, Danville, PA, United States

**Keywords:** SARS-CoV-2, COVID-19, stroke, pathophysiology, pharmacology, neuroimmunomodulation, renin-angiotensin system, blood coagulation

## Abstract

Since the early days of the pandemic, there have been several reports of cerebrovascular complications during the severe acute respiratory syndrome coronavirus 2 (SARS-CoV-2) infection. Numerous studies proposed a role for SARS-CoV-2 in igniting stroke. In this review, we focused on the pathoetiology of stroke among the infected patients. We pictured the results of the SARS-CoV-2 invasion to the central nervous system (CNS) via neuronal and hematogenous routes, in addition to viral infection in peripheral tissues with extensive crosstalk with the CNS. SARS-CoV-2 infection results in pro-inflammatory cytokine and chemokine release and activation of the immune system, COVID-19-associated coagulopathy, endotheliitis and vasculitis, hypoxia, imbalance in the renin-angiotensin system, and cardiovascular complications that all may lead to the incidence of stroke. Critically ill patients, those with pre-existing comorbidities and patients taking certain medications, such as drugs with elevated risk for arrhythmia or thrombophilia, are more susceptible to a stroke after SARS-CoV-2 infection. By providing a pictorial narrative review, we illustrated these associations in detail to broaden the scope of our understanding of stroke in SARS-CoV-2-infected patients. We also discussed the role of antiplatelets and anticoagulants for stroke prevention and the need for a personalized approach among patients with SARS-CoV-2 infection.

## Introduction

As a member of the coronavirus family, severe acute respiratory syndrome coronavirus 2 (SARS-CoV-2), the etiological agent of coronavirus disease of 2019 (COVID-19), is an enveloped virus with a positive-sense single-stranded RNA genome, that exhibits ~80 and 50% genetic similarity with severe acute respiratory syndrome coronavirus 1 (SARS-CoV-1) and Middle East respiratory syndrome coronavirus (MERS-CoV), respectively (1, 2). While the pathogenesis of SARS-CoV-2 remains to be clarified, its similarities with SARS-CoV-1 and MERS-CoV pathogenesis may provide insights into SARS-CoV-2 pathogenesis. With few exceptions, coronaviruses are generally associated with respiratory infections. Reports of a wide range of neurologic symptoms including stroke (3, 4), in addition to reports of virus detection in the cerebrospinal fluid (CSF) (5) and brain tissues from autopsies (6–8) introduced a neuroinvasive potential of SARS-CoV-2. Although a causal relationship between coronaviruses and stroke has not yet been established, supporting evidence exists in several publications. First, there is an independent association between COVID-19 and acute ischemic stroke after controlling for other vascular risk factors (9). Further, in a study of 17,799 hospitalized patients with SARS-CoV-2 infection, our team reported a pooled stroke risk of 0.9% while ischemic stroke occurred in 79% of patients, hemorrhagic stroke in 17%, and 4% had cerebral venous thrombosis (10). Other meta-analyses reported a stroke incidence rate of 1.1–1.6% among patients with COVID-19 (11–14) which appears higher than 0.6–0.8% incidence in the general population (15). Evidence has shown a significant decrease in the rate of myocardial infarction and ischemic stroke in the emergency department during the pandemic (16). This phenomenon, which may be attributable to the fear of SARS-CoV-2 infection among the population, suggests the underdiagnosis of stroke and a possible higher incidence of stroke in SARS-CoV-2 infected patients. Moreover, the risk of stroke in SARS-CoV-2 infection is 7.6-fold higher than that of influenza infection (17). Finally, cerebrovascular diseases were an independent predictor of severity and fatality of COVID-19 illness based on adjusted effect estimates (18).

Moreover, stroke in SARS-CoV-2 infected patients is reported to have specific features. It is more commonly reported in young patients (mean age <55 years) without classic vascular risk factors (19–21), with a high prevalence of cryptogenic stroke (11, 22), and an increased incidence of large vessel stroke (11, 21, 23, 24), even in patients with mild SARS-CoV-2 infection (25). These reports highlight the importance of acknowledging the association between stroke and SARS-CoV-2 infection.

Given the former considerations, we conducted a narrative review of possible pathways responsible for neuroinvasion by SARS-CoV-2. We also provided a pictorial overview of the topic to better summarize the potential etiopathogenic mechanisms underlying the stroke in patients with SARS-CoV-2 infections.

## SARS-CoV-2 Cellular Entry

**Figure 1:**
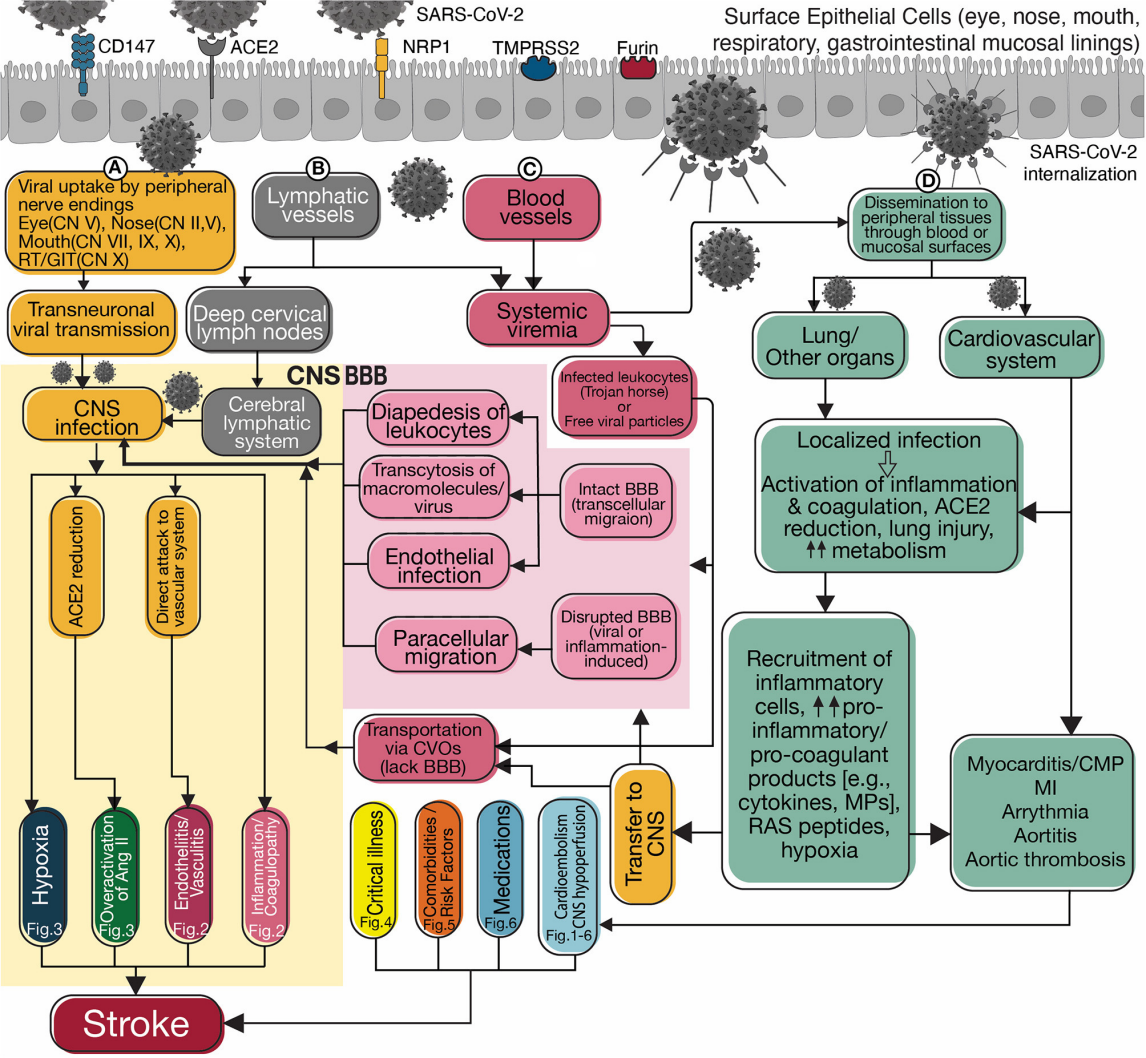
Flowchart diagram summarizes possible routes for neuroinvasion and potential mechanisms of stroke. SARS-CoV-2 penetrates through its receptors, ACE2, CD147, or NRP1 and co-receptors, TMPRSS2, or FURIN present on the epithelial cells of the surface mucosal lining. Transportation occurs via viral invasion into **(A)** peripheral nerve endings and transneuronal transmission, **(B)** peripheral lymphatic vessels connected to the blood or cerebral lymphatic circulation, and **(C)** the blood vessels and invasion into the CNS (yellow box on the left). In blood, the virus spreads inside infected leukocytes (aka “Trojan horse”) or as free particles. Viral invasion through the hematogenous route requires crossing the intact blood-brain barrier (BBB) or penetrating the CNS through circumventricular organs (CVOs) that lack BBB. Intact BBB may allow a limited number of leukocytes to enter the CNS via transcellular diapedesis. Viral particles and macro-molecules can breach the BBB via receptor-mediated transcytosis or adsorptive transcytosis. Finally, SARS-CoV-2 can directly infect the endothelium and cross. A disrupted BBB enables unlimited paracellular transportation. Once In the CNS, the virus can invade the vascular system and increase stroke risk by causing endotheliitis/vasculitis (Figure 2), activating the inflammation and coagulation cascades (Figure 2), overactivation of angiotensin II signaling pathways (Figure 3), or causing local hypoxia (Figure 3). There is crosstalk between the inflammatory and coagulation systems in the central and peripheral environments because their products are transported bidirectionally. Viral invasion into peripheral tissues, including pulmonary and cardiovascular systems, occurs through direct mucosal surfaces or hematogenous dissemination **(D)**. Activation of inflammation and coagulation systems increases pro-inflammatory and pro-coagulant products, which are then transported to the CNS accompanying angiotensin II through BBB and CVOs. Lung and cardiovascular injury and elevated metabolism cause systemic hypoxia increasing the risk of stroke. Cardiovascular damage (Figures 1–6) can cause a stroke by arrhythmia and cardioembolism or by decreased cardiac output resulting in cerebral hypoperfusion. Clinical features of critical illness (Figure 4), especially in patients with some comorbidities or risk factors (Figure 5) and side effects of certain medications (Figure 6) may be related to increased risk of stroke. SARS-CoV-2, Severe acute respiratory syndrome coronavirus 2; CD147, Cluster of differentiation 147; ACE2, Angiotensin-converting enzyme 2; NRP1, Neuropilin-1; TMPRSS2, Transmembrane Serine Protease 2; CN, Cranial Nerve; RT, Respiratory tract; GIT, Gastrointestinal tract; CNS, Central nervous system; Fig, Figure; Ang II, Angiotensin 2; BBB, Blood-brain barrier; CVOs, Circumventricular organs; MPs, Microparticles; RAS, Renin-angiotensin system; CMP, Cardiomyopathy; MI, Myocardial infarction.

The main transmission route for SARS-CoV-2 is the direct contamination of mucosal linings (26) (Figure 1). Epithelial cells in the mucosal linings of the respiratory and gastrointestinal tracts can spread the virus by expressing the main receptor of SARS-CoV-2—angiotensin-converting enzyme-2 (ACE2)—and the main cofactor—transmembrane serine protease 2 (TMPRSS2) (27). TMPRSS2 is an activating protease that is necessary for cleaving the spike protein of SARS-CoV-2, facilitating the viral binding to the receptor, and leading to viral internalization (28). In addition to ACE2, the cluster of differentiation 147 (CD147), which acts through interaction with spike glycoprotein, was introduced as a novel receptor for SARS-CoV-2 (29). A furin cleavage site, which was not present in the spike glycoprotein of SARS-CoV-1, has also been discovered in SARS-CoV-2 as a host factor participating in viral infection (30). Furin is a ubiquitous protease that participates in activating surface proteins of viruses such as coronaviruses and influenza (31, 32). Although not consistently expressed in all cell types, ACE2, TMPRSS2, CD147, and furin are present in the human respiratory tract (33). Furthermore, neuropilin-1 (NRP1), a cellular transmembrane receptor, has been introduced to act as a specific receptor for SARS-CoV-2 (34, 35). Epithelial cells of respiratory and gastrointestinal linings highly express NRP1 (34). NRP1 has been shown to mediate viral infection even in the absence of TMPRSS2 and ACE2. However, the infection is milder compared to the sole expression of ACE2 (34).

After passing through the mucosal linings, the systemic spread of SARS-CoV-2 to tissues, blood and lymphatic circulation, and peripheral nerve endings can enable the spread to the central nervous system (CNS). The brain is privileged by having various barriers such as the blood-brain barrier (BBB) and the blood-cerebrospinal fluid barrier (36). However, the integrity of these barriers is not complete and pathologic conditions may disrupt them (37). After crossing the barriers, SARS-CoV-2 neuroinvasion needs the presence of viral receptors in the host brain cells. Human brain cell lines express ACE2, CD147, NRP1, and TMPRSS2 (38, 39).

### Proposed Mechanisms for Neuroinvasion

Two possible routes can be hypothesized for SARS-CoV-2 neuroinvasion, including neuronal and hematogenous transmission. Figure 1 pictures the possible mechanisms of stroke associated with SARS-CoV-2 in each of these routes.

#### Neuronal Route of Neuroinvasion

Transneuronal transport of the SARS-CoV-2 through peripheral nerve endings can occur via specific viral receptors (40) (Figure 1A). In addition, peripheral nerve endings may use other mechanisms for uptake of the virus. For example, viral uptake can occur via trans-synaptic membranous-coating-mediated endocytosis (similar to other coronaviruses) (41) or fusion of virus envelope with the host neuron via the axonal membrane of the next-order-neuron (similar to herpesviruses) (42).

Like other mucosal linings, nasal mucosa (the respiratory mucosa and olfactory mucosa) may be an entry site for SARS-CoV-2. The human nasal mucosa and olfactory bulb express different levels of NRP1, ACE2, CD147, TMPRSS2, and Furin, which may explain the smell and taste disturbance (34, 43–45). The expression of ACE2 and TMPRSS2 was not obvious in the neurons of the olfactory mucosa and olfactory bulb, however, they were detected in the non-neuronal cells (such as support cells, stem cells, and perivascular cells) (43). Neuropathological analysis of COVID-19 deceased patients showed high expression of NRP1 in SARS-CoV-2 infected cells of the olfactory epithelium, and tracts (34).

The neuroinvasive potential of respiratory coronaviruses through the olfactory nerve was previously examined in mice models inoculated with intranasal SARS-CoV-1. Viral antigens were primarily detected in the olfactory bulb, areas of the brain connected directly to the olfactory bulb, and then dissemination throughout the brain (46). Further studies on the autopsy of a COVID-19 patient with anosmia, dysgeusia, and seizure revealed diffuse tissue damage in the olfactory pathway, rectus gyrus, and medulla oblongata. Neurons, glial cells, axons, and nerve sheaths were damaged, and particles resembling virions of SARS-CoV-2 were detected in these structures (47). In another study, asymmetric olfactory bulb with or without olfactory cleft obliteration was present in 4 out of 19 early postmortem MRI examinations of patients who died from COVID-19; however, downstream olfactory tract changes were not evident (48). Moreover, a recent MRI study reported that persistent anosmia in COVID-19 patients evaluated with objective olfactory tests may be associated with olfactory bulb atrophy (49).

Intranasal inoculation of murine coronavirus resulted in widespread infection, including the detection of viral genome in the trigeminal and olfactory nerves, and areas in the brain connected to them (50). Observing this pattern of viral spread by following known neuroanatomical pathways supports trans-neural transmission through this nerve (50). Trigeminal nerve involvement was proposed as a possible mechanism of headache in COVID-19 patients (51).

ACE2 and TMPRSS2 are present in the human conjunctiva and the cornea (52). Ocular manifestations (53) and SARS-CoV-2 presence in tears and conjunctival secretions (54) were reported. Eyes may provide an entry for SARS-CoV-2 through the trigeminal nerve into the CNS.

The involvement of trigeminal, facial, glossopharyngeal, and vagus nerves in the nasal and oral mucosa, which are responsible for the detection and transport of taste signals, maybe the reason for a high incidence of taste disturbance in the COVID-19 patients up to 88% (55, 56). As presented in animal models of influenza virus, viral spread to reach the brain via the vagus nerve is possible (57). The virus may hypothetically invade the vagus nerve in respiratory and gastrointestinal tracts and retrogradely infect the CNS (58).

#### Hematogenous Route for Neuroinvasion

Infection of mucosal linings may provide access to the lymphatic system (Figure 1B) and the bloodstream (Figure 1C) as endothelial cells express the SARS-CoV-2 receptors (59, 60). These pathways may disseminate the virus to the peripheral tissues such as the lungs and cardiovascular system (Figure 1D), or end up in the CNS. The brain has a functional lymphatic pathway connected to the deep cervical lymph nodes which is capable of transferring fluid and immune cells (61). The lymphatic pathway can be hypothesized to be an entry for the SARS-CoV-2.

BBB tightly protects the CNS micro-environment. However, peripheral leukocytes can minimally cross the BBB via transcellular diapedesis (37). During an inflammation, damaged tight junctions of endothelial cells make trafficking via paracellular routes possible (37). SARS-CoV-2 might cross the intact BBB and cause BBB damage directly or through induction of pro-inflammatory cytokines (e.g., IFN-γ) and chemokines (62). A monolayer culture of human endothelial cells showed low ACE2 levels. However, ACE2 upregulation has been noted due to shear stress in the 3-dimensional model of the middle cerebral artery, particularly in stenotic portions. In this model, recombinant SARS-CoV-2 spike protein attachment to ACE2 of endothelial cells induced various gene expression including the offending proteins in COVID-19 such as IL-4, IL-10, and complement C3. These findings further support the susceptibility of human brain endothelial cells to SARS-CoV-2 infection (63). Moreover, a model of human BBB showed disruption of endothelial barrier after introduction of SARS-CoV-2 spike protein via inducing a pro-inflammatory response on brain endothelium. This model added another evidence for neuroinvasive nature of the virus (64). Investigation of cadavers revealed SARS-CoV-2 particles packed in vesicles capillary endothelial cells and the neurons of the frontal lobe (6). Exocytosis and endocytosis of viral particles were also observed in endothelial cells which support the hematogenous route for viral invasion (6).

SARS-CoV-2 invasion to the lung appears to happen directly through viral disruption of alveolar and bronchial epithelial cells and macrophages, and indirectly via systemic inflammatory mediators. The type II alveolar epithelial cells and macrophages found in alveoli and pulmonary hilum lymphoid tissues are infected by SARS-CoV-2. COVID-19 patients with severe signs of pneumonia and acute respiratory distress syndrome (ARDS) have characteristics of systemic hyper-inflammation known as “cytokine storm” with exaggerated production of pro-inflammatory cytokines and chemokines, and also pro-coagulant factors (1, 65). Infection of respiratory epithelial cells, dendritic cells, and alveolar macrophages by SARS-CoV-2 might drive the cytokine storm (1, 65, 66). Pro-inflammatory products might result in damages in pulmonary tissues, and transfer of these products in the systemic circulation to other organs and failure of multiple organs such as cardio- and cerebrovascular systems. Mast cells, which are found in the submucosa of the respiratory system, are also hypothesized to have a crucial role in SARS-CoV-2 hyper-inflammation by releasing histamine and proteases and pro-inflammatory cytokines and chemokines (67). Through various mechanisms such as the expression of ACE2, mast cells recognize SARS-CoV-2 and recruit immune cells (68). Hypoxia, an outcome of respiratory failure and elevated metabolism, and elevated levels of angiotensin 2 may also link the pulmonary infection with cardiovascular events and stroke. Indirect effects of peripheral infection via transportation of these local products to the CNS may trigger a stroke even in the absence of viral neuroinvasion.

Moreover, reallocation of SARS-CoV-2-containing macrophages migrating out of the lungs to other tissues such as CNS is plausible in the context of viral spread. Infecting the leukocytes and using them as a reservoir was known in SARS-CoV-1 and some other neuroinvasive viruses (69, 70).

In addition to disruption of BBB, circumventricular organs (CVOs) are a candidate for entry to the brain. CVOs are specialized sensory areas of the brain participating in fluid homeostasis, cardiovascular regulation, and energy balance. CVOs have fenestrated capillaries that make them windows for the entrance of blood-borne products and pathogens to the brain (71). CNS infection through CVOs has not been reported in humans; however, animal models have demonstrated CVOs as a route for pathogens to invade the CNS (72).

## Factors Associated With Stroke in Patients Infected With SARS-CoV-2

### Endothelial Cells

**Figure 2:**
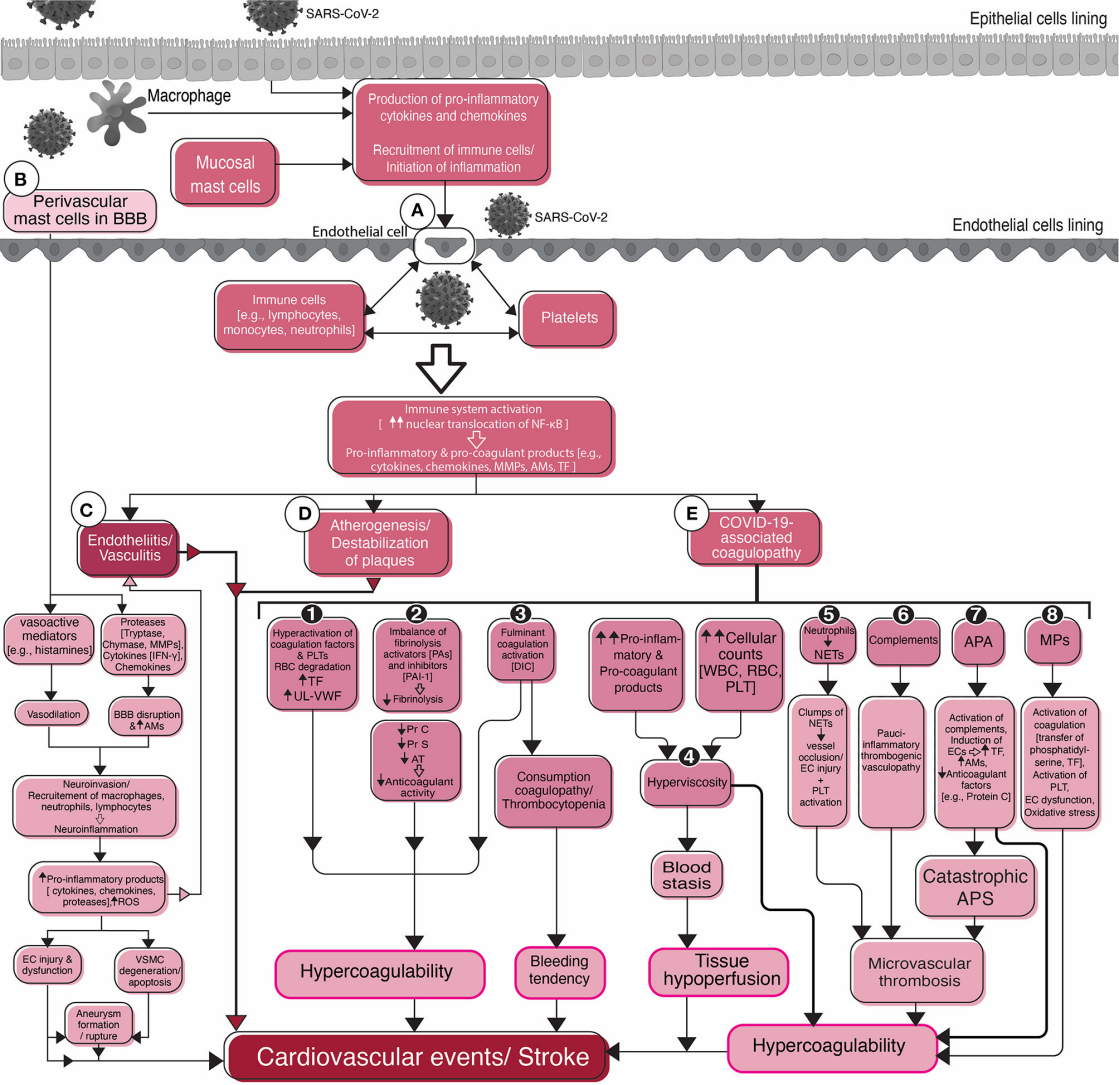
Flowchart diagram shows the roles of endothelial cells, platelets, the inflammatory system, and the coagulation system in SARS-CoV-2-associated stroke and cardiovascular events. Viral infection of epithelial cells of the mucosal linings stimulates them for the production of pro-inflammatory cytokines and activation of the immune system. Macrophages and mast cells residing in the mucosal tissues also participate in the activation of the immune system in the early stages of infection. Activation of and interaction between the immune system, endothelial cells, and platelets **(A)** in circulation result in stroke complications. Common sequelae include endotheliitis, or vasculitis **(C)**, atherogenesis, or destabilization of pre-existing atherosclerotic plaques **(D)**, and COVID-19-associated coagulopathy **(E)**. Different mechanisms that are attributable to COVID-19-associated coagulopathy are depicted in detail **(1–8)**. The possible role of mast cells in the blood-brain barrier (BBB) disruption, initiation and/or aggravation of inflammation, neuroinvasion, and stroke are summarized in the left side of the figure **(B)**. Mast cells, one of the two resident immune cells (microglia is the second one), cause BBB damage leading to the recruitment of peripheral immune cells and the virus into the brain and inflammation in the cerebral vasculature and subsequent stroke. SARS-CoV-2, Severe acute respiratory syndrome coronavirus 2; BBB, Blood-brain barrier; NF-κB, Nuclear factor-kappa B; MMPs, Matrix metallopeptidases; AMs, Adhesion molecules; TF, Tissue factor; IFN-γ, Interferon-gamma; ROS, Reactive oxygen species; EC, endothelial cell; VSMC, Vascular smooth muscle cell; PLT, Platelet; RBC, Red blood cell; UL-VWF, Ultra large-von Willebrand factor; PA, Plasminogen activator; PAI-1, Plasminogen activator inhibitor-1; Pr, Protein; AT, Antithrombin; DIC, Disseminated intravascular coagulation; WBC, White blood cell; NETs, Neutrophil extracellular traps; APA, Antiphospholipid antibodies; APS, Antiphospholipid syndrome; MPs, Microparticles.

Endothelial cells maintain vascular hemostasis and blood flow. Due to a large surface area, cells are exposed to intravascular signals from pathogens and inflammatory products (Figure 2C). Cells actively respond to stimuli by structural alterations, leading to increased vascular permeability and secretion of the pro-inflammatory cytokines. Cytokines recruit immune cells, such as neutrophils and platelets, and combine with pro-coagulant factors to form a thrombus that encases pathogens (73). Activated endothelial cells also influence the balance of vascular tone, resulting in diminished blood flow and ischemia (74). In addition to chemical signals from the virus, inflammatory cells, and platelets, direct viral invasion to endothelial cells also happens in SARS-CoV-2 infection. Diffuse endotheliitis, presence of viral particles in endothelial cells, and tissue infarction in various organs, including the lungs, heart, kidney, small intestine, liver (59), and brain (6), were reported in patients with SARS-CoV-2. Biomarkers associated with activated endothelial cells and platelets were significantly higher in SARS-CoV-2 infected ICU patients than non-ICU admitted patients (75). These markers were also associated with mortality, highlighting the prominent role of endotheliopathy in COVID-19 (75).

Viral-induced vasculopathy was reported in different viruses and may be a predisposing factor for stroke in SARS-CoV-2 infection as well. For instance, the varicella-zoster infection that has been associated with vasculopathy of large and small vessels may induce transmural involvement of the vessels, which in turn leads to ischemic and hemorrhagic strokes (76). SARS-CoV-1 infections are associated with systemic vasculitis, featuring leukocyte infiltration and endothelial cell proliferation, swelling, and apoptosis (77). Similarly, there are reports of patients with SARS-CoV-2 presenting with cerebral vasculitis and vasculopathy with micro- and macrohemorrhage (48, 78–80).

### Platelets

Platelets can restrict pathogens by releasing pro-coagulant and pro-inflammatory mediators, forming thrombus, and directly interacting with pathogens, endothelium, and immune cells (81) (Figure 2). Thrombocytopenia is a common complication of viral infections, with diverse etiologies including SARS-CoV-2, (82) and may increase bleeding tendency. Thrombocytopenia and thrombocytosis were both associated with the severity and mortality of COVID-19 (83, 84). Thrombocytosis may be induced by thrombopoietin, released in the presence of pro-inflammatory cytokines such as IL-6 (85). Platelet count elevation was reported as a distinctive feature in SARS-CoV-2-induced pneumonia vs. other etiologies (86). There is a low risk of venous and arterial thrombus formation following reactive thrombocytosis (87, 88). In a group of hospitalized SARS-CoV-2 patients, platelet counts >450 × 10^9^/L, were predictive of thrombotic events and platelet counts below 150 × 10^9^/L were predictive of hemorrhagic events (89).

### Inflammatory System

Direct activation of the immune system, platelets, and endothelial cells by SARS-CoV-2 appears to play a pivotal role in further activation of the inflammatory system and chemical release (Figure 2A). The exaggerated inflammatory responses may cause vascular events including stroke through various mechanisms that are being further discussed.

#### Mast Cells

Mast cells that reside in the cerebral perivascular spaces and are presumed to be the first responders in inflammatory reactions in the CNS have a key role in the disruption of BBB (90) (Figure 2B). Mast cells produce various types of vasoactive mediators such as histamine, proteases (e.g., tryptase and chymase), cytokines, and chemokines. Release of these products causes endothelial dysfunction, BBB disruption, expression of adhesion molecules leading to leukocyte trafficking to the affected tissue, and neuroinflammation (90, 91). Therefore, they may be implicated in the initiation or deterioration of stroke. Activated mast cells participate in the pathogenesis of ischemic and hemorrhagic stroke, and hemorrhagic transformation of ischemic stroke after treatment with recombinant tissue plasminogen activator (92). Release of vasodilatory and proinflammatory substances, damage to the BBB, recruitment of immune cells, and neuroinflammation have been related to the role of mast cells in stroke (92, 93). Although it has not been observed in the CNS, degranulating mast cells were observed in the vessel wall of an infarcted spleen associated with SARS-CoV-2 vasculitis (94). Moreover, experimental models revealed the role of mast cells in the pathogenesis of cerebral aneurysm via induction of inflammation in the vessel wall. The inhibition of activated mast cells successfully prevented the progression of an aneurysm (95). Human studies confirmed abundant mast cells in the cerebral aneurysm wall and significant elevation in ruptured aneurysms (96). The formation of a cerebral aneurysm was also associated with the activation of macrophages, neutrophils, and leukocytes with the production of pro-inflammatory cytokines and matrix metalloproteinases (MMPs). In addition, endothelial dysfunction and vascular smooth muscle cell degeneration, and apoptosis leading to vessel wall thinning were also linked with cerebral aneurysm (97).

#### Atherogenesis and Destabilization of Plaques

Infection increases pro-inflammatory cytokines that in turn activate inflammatory cells inside the atherosclerotic plaques (98) (Figure 2D). Cytokines and intraplaque inflammatory cells destabilize the preexisting atherosclerotic plaques via increasing proteins such as metalloproteinases and oxidative stress (98, 99). Moreover, hyper-secretion of cytokines and chemokines during SARS-CoV-2 infection are hypothesized to stimulate the formation of new-onset vascular plaques (100). Inflammatory products cause oxidative stress, damage the endothelium and overlying fibrous cap of atherosclerotic plaques, activate the platelets and stimulate the vascular smooth muscle cells to migrate into the intima, and produce fibrous products which lead to the production of fatty streaks (100). In addition, acute infection and inflammation increase pro-coagulant products and cause a hypercoagulable state. Arterial surface breakdown at the site of plaque rupture in the presence of a hypercoagulability state increases the risk for acute thrombogenesis (98, 99).

#### COVID-19-Associated Coagulopathy

Although not completely understood, COVID-19-associated coagulopathy (CAC, Figure 2E) shares characteristics with sepsis-induced coagulopathy, disseminated intravascular coagulopathy (DIC), or thrombotic microangiopathies (101). Nonetheless, CAC has distinctive features, such as a higher incidence of thrombotic events than sepsis, the prominent role of inflammatory cytokines, complements, and antiphospholipid antibodies (101). Moreover, despite frequent thrombotic events in severe COVID-19, milder thrombocytopenia, and milder prolongation of prothrombin time (PT) compared to sepsis-induced DIC occurs (101). Important characteristics of CAC that may be hypothesized to stimulate cardiovascular events and stroke are explained.

Direct damage and activation of endothelial cells by SARS-CoV-2 may initiate a procoagulant state, as it occurs during sepsis (73). Direct interaction with platelets and inflammatory cells, and secretion of pro-coagulant factors, such as tissue factor (TF) and von Willebrand factor (vWF), follows. Furthermore, activation of the coagulation cascade, inhibition of the anticoagulation system and fibrinolysis, and change in blood flow are associated with inflammation-induced coagulopathy (73) (Figure 2-E1, E2). Inflammatory cytokines may induce endothelial cells to secrete hyperactive ultra-large vWF multimers and prevent the cleavage of vWF to less active fragments (102). Furthermore, in patients with COVID-19, hyperactivation of the fibrinolysis system has been reported due to endothelial injury, elevated plasminogen activators, and elevated D-dimer levels (103). However, despite the elevated D-dimer levels, which usually suggest a hyperfibrinolytic state, a paradoxical fibrinolysis shutdown was shown in patients with COVID-19 (104). This hyperfibrinolytic state is not expected in sepsis-induced coagulopathy or DIC and is unique in COVID-19. The counter-balance of hyperfibrinolysis by elevated levels of fibrinolytic inhibitors, such as plasminogen activator inhibitor-1 (PAI-1), may in part explain the frequent thrombotic events in SARS-CoV-2 infection (105).

**Figure 3:**
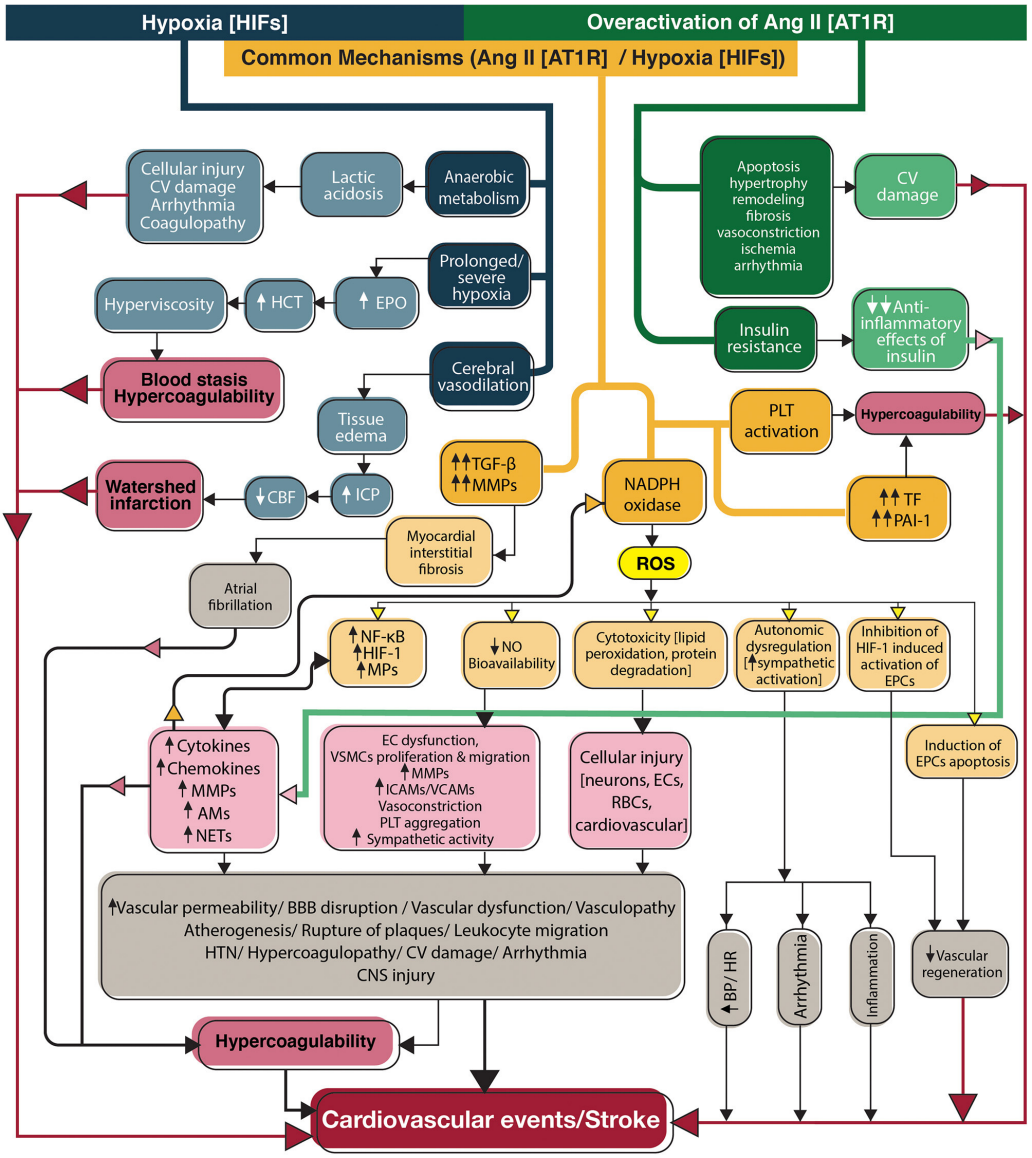
Flowchart diagram shows the contribution of the renin-angiotensin system and hypoxia to stroke in SARS-CoV-2 infection. Overactivation of angiotensin II signaling pathways and hypoxia directly activate inflammation and coagulation systems, in addition to indirect activation through induction of NADPH, to produce reactive oxygen species (yellow box). Ang II acts through its main receptor, angiotensin type 1 receptor, and hypoxia mediates its effects through transcription factors, hypoxia-inducible factors. CV, Cardiovascular; EPO, Erythropoietin; HCT, Hematocrit; ICP, Intracranial pressure; CBF, Cerebral blood flow; TGF-β, Transforming growth factor-beta; MMPs, Matrix metallopeptidases; NADPH, Nicotinamide adenine dinucleotide phosphate; ROS, Reactive oxygen species; PLT, Platelet; TF, Tissue factor; PAI-1, Plasminogen activator inhibitor-1; HIFs, Hypoxia-inducible factors; NF-κB, Nuclear factor-kappa B; HIF-1, Hypoxia-inducible factor-1; MPs, Microparticles; AMs, Adhesion molecules; NETs, neutrophil extracellular traps; NO, Nitric oxide; EC, Endothelial cell; VSCM, Vascular smooth muscle cell; ICAMs, Intercellular adhesion molecules; VCAMs, Vascular cell adhesion molecules; BBB, Blood-brain barrier; HTN, Hypertension; CNS, Central nervous system; RBCs, Red blood cells; BP, Blood pressure; HR, Heart rate; EPCs, Endothelial progenitor cells; Ang II, Angiotensin II; AT1R, Angiotensin type 1 receptor; HIFs, Hypoxia-inducible factors.

An abnormal elevation of plasma macromolecules such as immunoglobulins and fibrinogen in addition to cellular elements (erythrocytes, leukocytes, or platelets) increase the blood viscosity, impairs blood flow in the microvasculature, and damages endothelium (106, 107). Hyperviscosity is associated with hypercoagulability, blood stasis, and tissue ischemia complications (106, 107) (Figure 2-E4, Figure 3). COVID-19-associated hyperviscosity has been reported in patients with thrombotic complications which may be linked with hyper-fibrinogenemia or exaggerated cytokine release in SARS-CoV-2 infection (108).

Neutrophils secret an extracellular network consisting of proteolytic enzymes and chromatin (Figure 2-E5). The network, which attacks local pathogens, are called neutrophil extracellular traps (NETs). If uncontrolled, NETs cause cellular damage, endothelial injury, thrombosis, or vascular rupture and hemorrhage and they may have a pivotal role in the pathogenesis of severe COVID-19 (109). Plasma NETs are associated with disease severity in COVID-19 patients and micro-thrombosis through interaction with platelets (110). By forming intravascular clumps, NETs occlude the vessels, and accelerate thrombus formation leading to micro-vasculopathy in COVID-19 (111). The association between plasma NETs in patients with acute stroke and the severity of the stroke suggests the prominent role of neutrophils in arterial thrombosis (112). Neutrophils and NETs were detected in cerebral clots that were retrieved during endovascular reperfusion therapies from patients with large vessel occlusions. The specimens showed successful *ex vivo* thrombolysis after adding DNase-1 (to disrupt NETs) to tissue plasminogen activator (t-PA) (113).

Complement proteins are parts of the innate immunity that are implicated in coagulopathy and thrombosis (114) (Figure 2-E6). Pathologic examination of lung tissue and purpuric skin lesions of five patients with severe COVID-19 showed pauci-inflammatory thrombogenic vasculopathy with complement deposition (115). Based on data describing the co-localization of SARS-CoV-2 spike glycoproteins with complement products in these patients, direct activation of complement was hypothesized (115). Other evidences support that complement-induced endothelial injury is a chief contributor to the development of inflammation, thrombotic events, and organ failure in COVID-19 (116). SARS-CoV-2 spike protein subunits seem to directly activate the alternative complement pathway on the cellular surface (117). Attachment of SARS-CoV-2 spike protein to endothelial cells upregulates the expression of C3 (63). C3 reduction, due to overactivation and conversion to active components, was related to the poor prognosis of COVID-19 patients (118). Besides, elevated levels of activated complement products have been associated with the biomarkers of endothelial damage and the severity of COVID-19 (119).

Secondary antiphospholipid antibody syndrome (APS) may explain stroke in relatively young patients with COVID-19 (120). Elevation of antiphospholipid antibodies (APA) (Figure 2-E7) is associated with a higher incidence of thromboembolic events during viral infections (121). In a cohort of ICU-admitted COVID-19 patients with frequent thrombotic complications, positive APA was reported in 88% of patients (122). Multi-territory ischemic stroke was also reported in patients with COVID-19 and positive APA (123). Increased expression of adhesion molecules by endothelium, induction of TF, inhibition of endogenous anticoagulants in the protein C pathway, and activation of the complement system have been suggested as causes for APA-related thrombosis (124). Catastrophic APS is a rare entity and a severe form of APS with a high mortality rate. It causes microvascular thrombosis, rapid multi-organ dysfunction (125), and could be associated with the microvasculopathy observed in some severe COVID-19 infections.

Microparticles (MPs) are plasma membrane-derived vesicles blebbing during activation or apoptosis of different cell types, mainly including platelets, endothelial cells, and leukocytes. MPs present antigens similar to their cell of origin (126) (Figure 2-E8). In pathologic conditions such as sepsis and vascular diseases, the circulating levels of MPs elevate and participate in inflammation, coagulation, vascular dysfunction, and oxidative stress (126). Platelet-derived MPs are implicated in acute ischemic stroke (127). Their pro-coagulant activity is mainly associated with the presence of phosphatidylserine in the outer cell surface, providing a catalytic surface for the assembly of coagulation factors and thrombin generation and expression of TF (128). Higher levels of pro-coagulant MPs were detected in hospitalized COVID-19 patients compared to patients without the disease (129). Circulating MPs are correlated with the expression of pro-inflammatory cytokines in patients with COVID-19 and a possible predictor of the severity of COVID-19 (130).

## Classic and Alternative Renin-Angiotensin System

The renin-angiotensin system (RAS) is primarily responsible for regulating blood pressure, water, and electrolytes (131) (Figure 3, green and orange pathways). Angiotensin II (Ang II) is produced from angiotensin I by the angiotensin-converting enzyme (ACE). Ang II is a potent vasoconstrictor and acts via its main receptor, angiotensin II receptor type 1 (AT1R), which is expressed in several tissues. Angiotensin II receptor type 2 (AT2R) with a lower affinity for Ang II counteracts with AT1R (131). On the other hand, ACE2, the homolog of ACE, is the main regulator of the classic RAS (132). ACE2 cleaves Ang II to Ang (1–7) and diverts the system to the alternative RAS with vasoprotective outcomes such as decreasing blood pressure, inflammation, and atherosclerosis. The main receptor of Ang (1–7) is Mas, but it also triggers AT2R. Ang (1–7) increases bradykinin levels by inhibiting ACE from its degradation and promotes vasodilation and fibrinolysis by bradykinin (132).

### Consequences of ACE2 Reduction and Angiotensin II Overactivation

Receptor-mediated endocytosis of SARS-CoV-2 with ACE2 causes an elevation in the ACE/ACE2 ratio (133). Overactivation of the classic RAS may increase the risk of both hemorrhagic and ischemic stroke (134). The brain has an independent RAS that does not interact with peripheral RAS because of the BBB (135). However, blood-borne Ang II enters the CNS across the disrupted BBB or the circumventricular organs (136).

Ang II stimulates vascular events with activation of inflammation and coagulation. AT1R inhibition was shown to be a useful immunosuppressive therapy (137). It directly activates toll-like receptors on the leukocytes to stimulate “nuclear factor kappa B” (NF-κB) and produce cytokines, chemokines, adhesion molecules, and metalloproteinases. It also damages the endothelium resulting in atherosclerosis, destabilization of plaques, and thrombosis. Ang II decreases the anti-inflammatory effects of insulin by inhibiting its signal transduction, further increasing inflammation (138). Ang II promotes coagulation via stimulation of tissue factor expression and platelet aggregation. Conversely, by increasing PAI-1 and decreasing t-PA via breakdown of bradykinin, Ang II inhibits fibrinolysis (139).

### Ang II and Oxidative Stress

Ang II downregulates the expression of nitric oxide (NO) by endothelium. NO is vasoprotective and prevents atherosclerosis by influencing endothelial cells, platelets, and vascular tone (140). NO is a potent endogenous vasodilator that counteracts with Ang II to increase regional blood flow and regulate BP. NO prevents coagulation and inflammation by inhibiting platelet aggregation and leukocyte adhesion (141). Ang II also decreases NO bioavailability by induction of nicotinamide adenine dinucleotide phosphate (NADPH) oxidase to produce reactive oxygen species (ROS) (142). The significant role of NADPH oxidase activity in disease severity was shown in COVID-19 as its activity was higher in patients than controls and was observed to be markedly elevated in patients who required ICU admission. It was also higher in patients with thrombotic events than patients without thrombosis independent from other vascular risk factors suggesting the implication of oxidative stress in COVID-19-associated coagulopathy (143). The role of ROS in Ang II signaling has been specifically observed in CNS (144). Pathologic ROS levels are cytotoxic and cause vascular oxidative stress involved in the pathogenesis of stroke (145). ROS trigger NETs production which in turn promote vascular events (146). Through oxidative stress, Ang II increases endothelial progenitor cell apoptosis and decreases re-endothelialization and vascular regeneration after injury (147).

### Cardiovascular Effects of Ang-II

The other well-established effect of Ang II is the enhancement of sympathetic activity, impacting cardiovascular responses such as heart rate and blood pressure (148). NO modulates this effect with anti-sympathetic activity (149). Ang II also promotes atherosclerosis and vascular dysfunction by creating oxidative stress. ROS stimulate vascular smooth muscle cell proliferation and migration (150) which elevate the risk for future cardiovascular and cerebrovascular events in SARS-CoV-2 infected survivors.

Cardiomyocytes undergo hypertrophy and remodeling with stimulation of AT1R (151). Through induction of transforming growth factor beta 1 and MMP-9 (associated with myocardial matrix remodeling) and induction of myocardial fibrosis, hypoxia-inducible factor 1α and Ang II are associated with pathogenesis and maintenance of atrial fibrillation (AF) (152).

## Hypoxia

Hypoxia was an independent prognostic factor for severity in patients with COVID-19. Hypoxia was associated with elevated white blood cell counts, C-reactive protein, and D-dimer levels in patients with COVID-19, possibly indicating a relationship between hypoxia, inflammation, and coagulopathy in severe infection (153). Hypoxia causes cerebral vasodilation and tissue edema by increasing substances like NO and prostacyclin (154). Continued or severe hypoxia causes anaerobic metabolism and lactic acidosis, which additionally enhances vasodilation and tissue edema (154). These events may increase intracranial pressure, limit the cerebral blood flow, and cause cerebral ischemia (Figure 3- blue and orange pathways).

### Role of Hypoxia-Inducible Factors

Hypoxia activates the expression of hypoxia-inducible factors (HIF1 and HIF2). These transcription factors have extensive crosstalk with the family of transcription factors NF-κB (155). Activation of inflammation requires nuclear translocation of NF-κB, which leads to the expression of the target genes. Enhanced activation of the NF-κB by HIFs is the key mechanism connecting hypoxia to inflammation (156). Hypoxia activates pro-coagulant factors such as TF by up-regulating the transcription factor early growth response-1 and restricts thrombolysis by increasing PAI-1 and reducing plasminogen activators (157). HIFs also increase NETs production that further promote thrombus formation (158). Platelets produce stabilized HIF-2α and PAI-1 and enhance thrombogenesis when stimulated by hypoxia (159). In addition, HIF-1-induced up-regulation of erythropoietin increases hematocrit, and blood viscosity and stimulates venous thrombosis (160).

### Hypoxia and Oxidative Stress

Hypoxia also stimulates of NADPH oxidase to increase ROS. ROS cause lipid peroxidation, endothelial injury, increased vascular permeability, and disruption of BBB, which may further enhance the tissue edema (161). HIF-1 and ROS are involved in the pathogenesis, progression, and rupture of atherosclerotic plaques and arterial thrombosis (162).

### Cardiovascular Effects of Hypoxia

Hypoxic cardiovascular injury results in lower cardiac output, which is further augmented by concomitant acidosis and elevated workload due to hypoxia-induced peripheral vasoconstriction (163). Hypoxia triggers cardiac arrhythmia, possibly due to the detrimental effects of anaerobic metabolism and ROS on the normal functions of ion channels. This association may highlight the role of antioxidants in preventing arrhythmia during hypoxic conditions (164).

## Cardiovascular Complications

**Figure 4:**
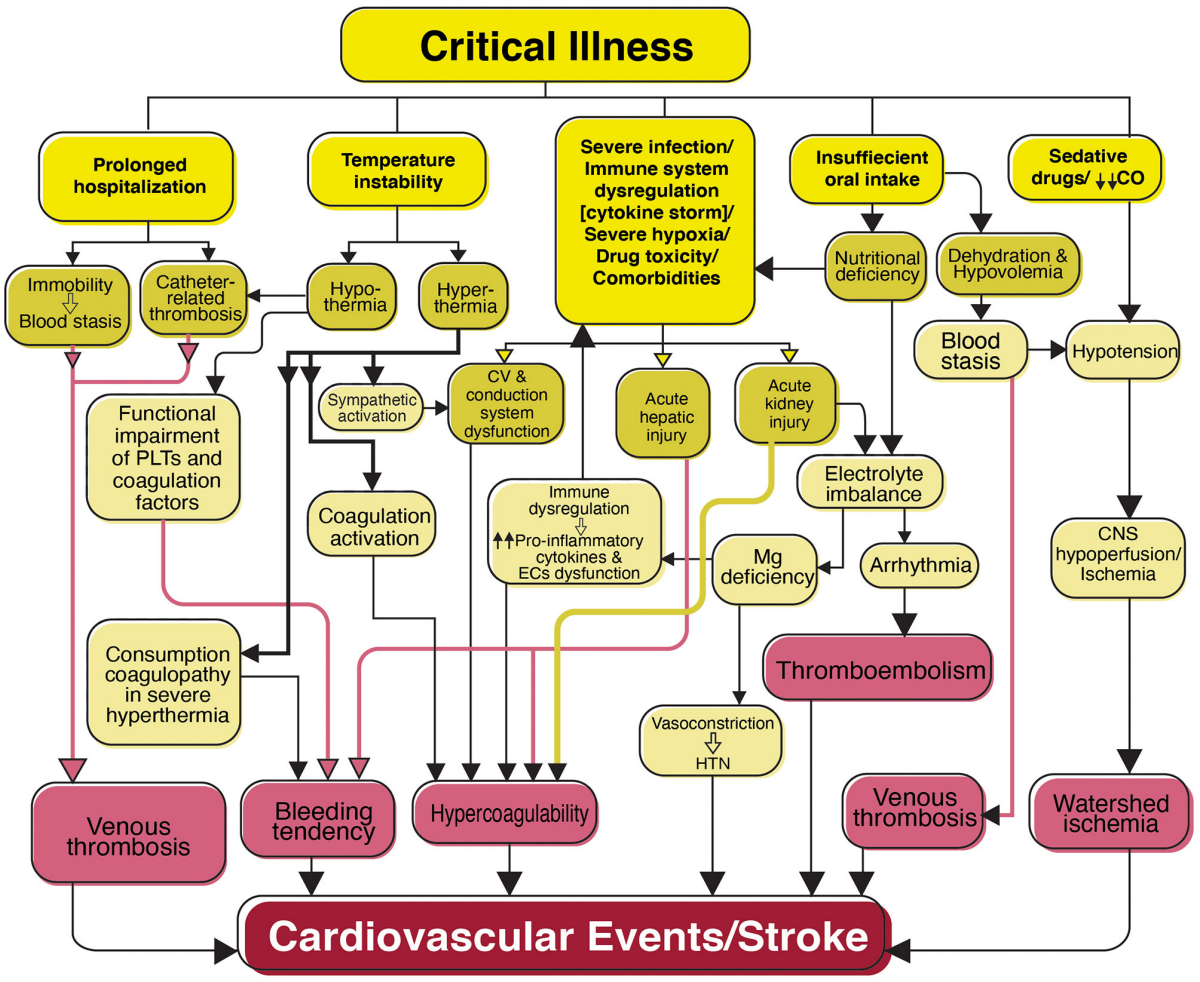
Flowchart diagram shows features of critical illness predisposing the patients with SARS-CoV-2 infection to cardiovascular events and stroke. Patients with a critical illness are mostly admitted to the intensive care unit (ICU) for prolonged periods. Clinical complications associated with critical illness including prolonged hospitalization, temperature instability, severe infection, and immune dysregulation, severe hypoxia, drug toxicity, comorbidities (e.g., diabetes mellitus, cardiovascular disease, and hypertension), insufficient oral intake, and sedative drugs are shown in association with their consequences that may result in cardiovascular events and stroke. PLTs, Platelets; CV, Cardiovascular; EC, Endothelial cells; Mg, Magnesium; HTN, Hypertension; CO, Cardiac output; CNS, Central nervous system.

**Figure 5:**
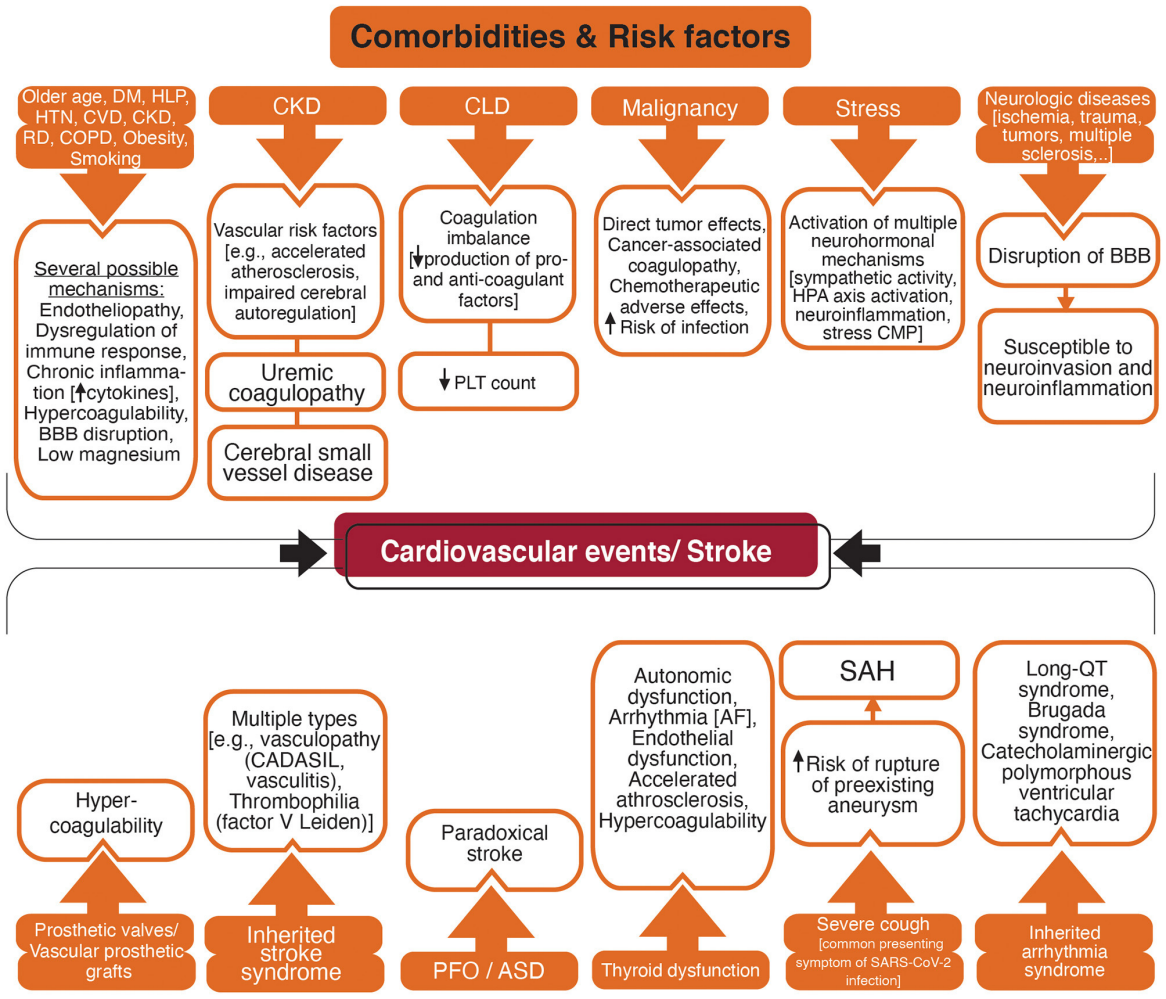
Flowchart diagram shows acquired and inherited factors with a higher risk for vascular events and possible associated mechanisms. DM, Diabetes mellitus; HLP, Hyperlipidemia; HTN, Hypertension; CVD, Cardiovascular disease; CKD, Chronic Kidney disease; RD, Rheumatic disease; COPD, Chronic obstructive respiratory disease; BBB, blood-brain barrier; CLD, Chronic Liver Disease; PLT, Platelet; HPA, Hypothalamic-pituitary-adrenal; CMP, Cardiomyopathy; CADASIL, Cerebral autosomal dominant arteriopathy with subcortical infarcts and leukoencephalopathy; PFO, Patent foramen ovale; ASD, Atrial septal defect; AF, Atrial fibrillation; SAH, Subarachnoid hemorrhage; SARS-CoV-2, Severe acute respiratory syndrome coronavirus 2.

**Figure 6:**
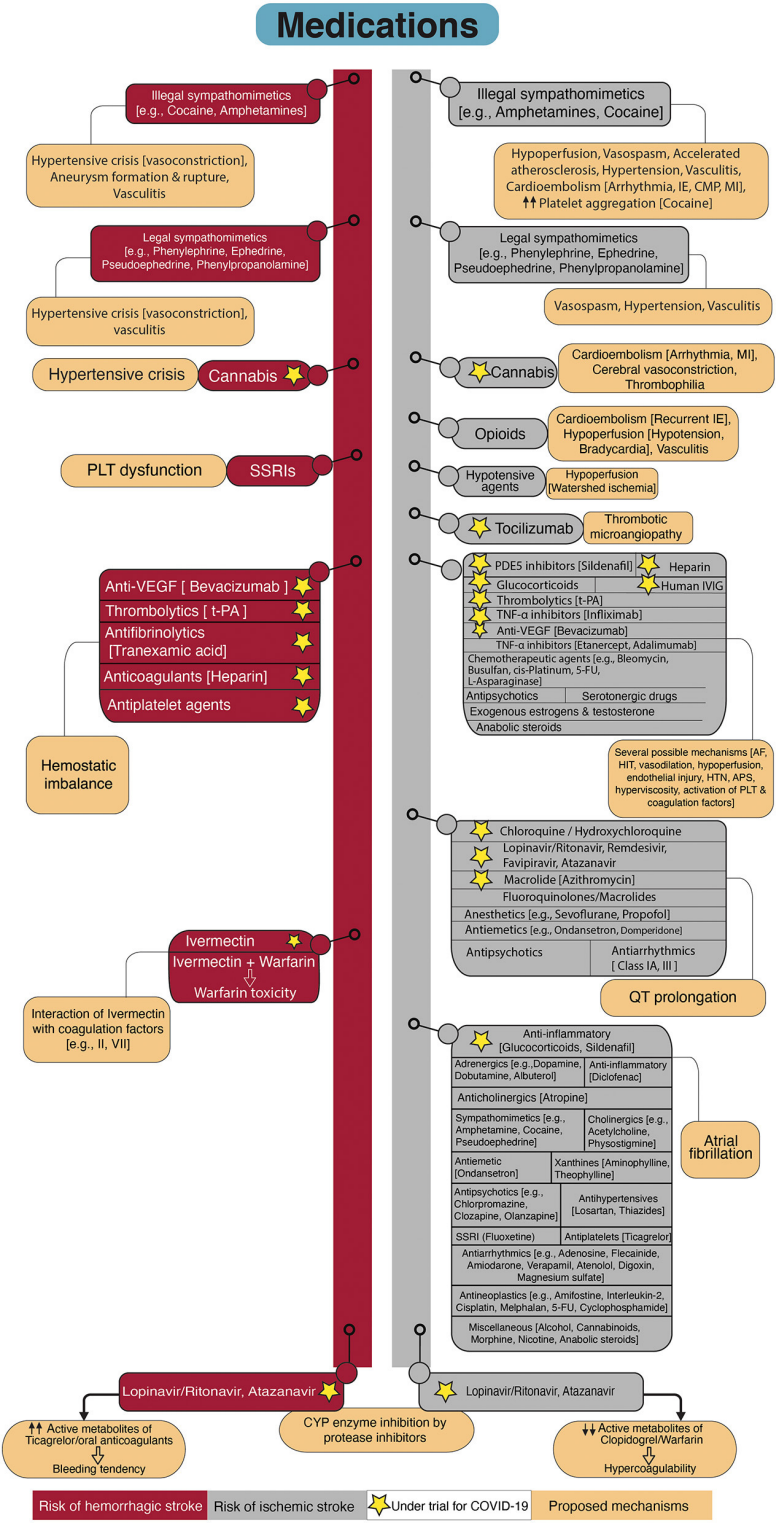
Flowchart diagram shows drugs associated with stroke and possible underlying mechanisms. The left side of the diagram represents drugs with elevated risk for hemorrhagic stroke. The right side of the diagram shows drugs that increase the risk for ischemic stroke. Proposed mechanisms of stroke related to the drugs are provided attached to each drug. Some of the drugs are under investigation for COVID-19 which are marked by asterisks. SSRIs, Selective serotonin reuptake inhibitors; PLT, Platelet; VEGF, Vascular endothelial growth factor; t-PA, Tissue plasminogen activator; CYP, Cytochrome P450; MI, Myocardial infarction; IE, Infective endocarditis; CMP, Cardiomyopathy; PDE5, Phosphodiesterase type 5; IVIG, Intravenous immunoglobulin; TNF-α, tumor necrosis factor-alpha; 5-FU, 5-Fluorouracil; AF, Atrial Fibrillation; HIT, Heparin-induced thrombocytopenia; HTN, Hypertension; APS, Antiphospholipid antibody syndrome.

Risk factors for cardiovascular complications in SARS-CoV-2 infection that increase stroke risk are shown in Figures 1–**6**. The incidence of acute cardiac injury was reported 15% by a meta-analysis of patients with COVID-19 (165). Among 138 hospitalized patients with SARS-CoV-2 infection, cardiovascular complications such as acute cardiac injury (7%) and arrhythmia (16.7%) were more prevalent in patients who required ICU admission (166). Another study of 416 hospital-admitted patients with SARS-CoV-2 reported cardiac injury in 20% of cases. Similarly, this study reported a higher rate of complications, such as coagulation disorders and mortality among patients with cardiac injury than patients without cardiac injury (167). Cardiovascular complications can increase the risk of stroke. Cardioembolic stroke accounted for 22% of ischemic stroke among 32 patients with SARS-CoV-2 and 15.7% in patients included in a meta-analysis (11, 22). On the other hand, it should be noted that COVID-19 shares common risk factors with cardiovascular and cerebrovascular diseases. Consequently, the association between SARS-CoV-2 infection and cardiovascular/cerebrovascular complications may not be causative and may only represent potential comorbidities (168).

Myocardial injury, can be a direct or indirect consequence of ischemic and non-ischemic insults to the myocardium. Cardiac muscles and vessels are potential targets for direct SARS-CoV-2 infection since ACE2 receptors have been detected in cardiomyocytes, vascular endothelial cells, pericytes -important in sustaining endothelium function-, and smooth muscle cells (169, 170). Previously injured hearts are more susceptible to direct damage due to upregulated ACE2 (170, 171). Direct invasion is supported by endomyocardial biopsy of infected patients with detection of the SARS-CoV-2 genome in five patients and endomyocardial damage due to the infiltration of immune cells (172). Direct injury of coronary arteries could cause macrovascular dysfunction, acute coronary syndrome, and myocardial infarction (MI). The other possible mechanism for ischemic myocardial injury is coronary microvascular dysfunction (CMD). CMD has various pathogenic mechanisms and despite the significant prevalence has diagnostic and treatment pitfalls (173). Endothelial cells are implicated as key contributors to both macro- and microvascular dysfunction in COVID-19; therefore, evaluation of the endothelial biomarkers has been suggested for risk assessment of COVID-19 patients (174). In addition to endothelial cells, direct injury of pericytes has been associated with COVID-19 microvasculopathy (170, 175). Pericytes express ACE2 abundantly and are considered major targets for SARS-CoV-2 in the heart (176) which may cause non-obstructive MI (177). Histological study of human lung specimens proposed the pivotal role of pericyte loss in COVID-19-induced microvasculopathy (178). Endothelial barrier dysfunction (as seen in diabetes or hypertension) is necessary for the virus to reach the pericytes. Based on the COVID-19-pericyte hypothesis, SARS-CoV-2 infected pericytes induce adjacent endothelium to release vWF and result in a pro-coagulant state (179). This finding suggests pericytes as a new therapeutic target for SARS-CoV-2. Takutsubo syndrome, which has been reported in patients with SARS-CoV-2 infection (180), could be another consequence of CMD (181).

Indirect injury can occur due to severe lung infection, an imbalance between oxygen supply and consumption, hyperinflammation, endothelial damage, and coagulopathy. Subsequently, they can induce various cardiovascular complications involving arrhythmia, myocarditis, hypertension, vasculopathy, and inflammation-induced atherogenesis or plaque instability leading to thrombosis and MI (177, 182). Additionally, Ang II further exacerbates the inflammation and coagulation which may aggravate cardiovascular complications. Atherosclerosis, hypertension, myocardial fibrosis, impaired contractility, and increased risk of AF are among the possible complications induced by Ang II (183).

Arrhythmia may be a consequence of direct viral invasion and subsequent myocarditis, or other viral infection complications, such as anxiety, sympathetic overactivity, arrhythmogenic cytokines, hypoxia and acidosis, and electrolyte disturbance, and medications (184). New-onset AF has been reported even as the presenting manifestation of the COVID-19 without clinical or imaging findings indicating COVID-19 pneumonia (184, 185). It was previously reported that sepsis-induced AF occurs in 6–20% of patients, and it increases the risk for ischemic stroke and death during hospitalization (186). Although the sepsis-induced AF is often transient, there might be a higher risk for 5-year AF recurrence and in-hospital and long-term stroke occurrence (187).

## Critical Illness

Critical illness and prolonged hospitalization raise the risk of strokes. Some of the factors associated with vascular events in patients with severe disease are presented in Figure 4.

Prolonged hospitalization causes immobility and increases the risk of venous thrombosis due to blood stasis and local hypoxia. Moreover, catheter-related thrombosis is a common complication of indwelling central venous catheters due to endothelial injury, blood stasis (188), and deep hypothermia (189).

Temperature instability in critical illness has been attributed to hemostatic disorders (190). Hyperthermia and decreased central blood volume have activated the sympathetic system and coagulation (showed by decreased activated partial thromboplastin time and elevated D-dimer) in the absence of concurrent sepsis, infection products, or evidence of endothelium injury (191). Although uncommon, there are reports of bleeding tendency due to consumption coagulopathy in severe hyperthermia (189). Despite the neuroprotective role of hypothermia in conditions such as brain ischemia (192), hypothermia-induced coagulopathy in ill patients may lead to hemorrhage by inhibiting platelet and coagulation factors (193).

Hepatic injury is one of the most common complications of COVID-19 and a predictor of disease severity and ICU admission (194). Hepatic injury was observed in the autopsy of COVID-19 patients (195). Pro-inflammatory cytokines, hypoxia, and drug-induced hepatotoxicity (194) in addition to direct viral invasion (196) are possible causes of liver injury in COVID-19. Considering the role of the liver in the coagulation system and drug metabolism, it is important to investigate the outcomes of COVID-19 liver injury, such as coagulation imbalance and risk for vascular events. As reported previously, other viruses such as cytomegalovirus, Epstein-Barr virus, and Hepatovirus A and B viruses have been related to thrombotic events in the setting of acute hepatitis (197). Although a causal relationship is not obvious, elevated liver tests are reported in patients who develop acute stroke in the course of SARS-CoV-2 infection (198, 199).

Up to 25% of critically ill patients with SARS-CoV-2 infection have been reported to develop acute kidney injury (AKI). Different mechanisms ranging from direct viral invasion to indirect effects such as RAS overactivation, hypovolemia, inflammation, hypercoagulability, and nephrotoxic drugs contribute to the development of AKI (7, 200). In addition to the risk of coagulation imbalance due to reduced medication excretion, AKI may also cause electrolyte disturbance and cardiac arrhythmia.

ICU patients with a critical illness are predisposed to nutritional deficiency due to insufficient oral intake and high metabolic demand. Electrolyte disturbance is one of the severe consequences of nutritional deficiency leading to arrhythmia and stroke. Magnesium has a significant role in immunomodulation via mediating many critical enzymatic reactions. By stimulating a pro-inflammatory state, increasing cytokine release, and endothelial dysfunction, hypomagnesemia may have an important role in COVID-19-associated coagulopathy (201). Magnesium supplementation has been used as an adjuvant treatment for COVID-19 patients and recommended for prevention and treatment, particularly in patients at risk for severe infection (202). Moreover, there is an association between hypomagnesemia and ischemic stroke due to vasoconstriction and hypertension (203).

Inadequate oral intake also leads to dehydration and hypovolemia that may predispose the CNS to hypoperfusion and ischemia, particularly in combination with cardiovascular dysfunction or administration of sedative medications that are frequently used among ICU patients. Hypovolemia also increases blood viscosity and stasis, predisposing patients to venous thrombosis.

## Comorbidities/Risk Factors

Figure 5 summarizes the possible comorbidities and risk factors associated with stroke in patients with COVID-19.

### Comorbidities With Proinflammatory and Procoagulant Phenotype

Pre-existing comorbidities such as older age, hyperlipidemia, diabetes, hypertension, cardiovascular disease, chronic kidney disease, chronic obstructive pulmonary disease (COPD), rheumatic diseases, malignancy, obesity, and smoking are related to the severe COVID-19 (204–206). These comorbidities are vascular risk factors with an increased risk for stroke in patients with SARS-CoV-2 infection (207). Endothelial dysfunction is a marker for atherosclerosis and is associated with vascular risk factors such as smoking, aging, hypercholesterolemia, hypertension, and hyperglycemia (208). Considering that endothelial cells are one of the main targets for SARS-CoV-2 (75), patients with pre-existing endothelial dysfunction are possibly more susceptible to endotheliopathy and vascular events. The chronic inflammatory state is a low-grade but long-term inflammation with elevated pro-inflammatory cytokines which occurs in conditions such as older age, stress, diabetes, hypertension, cardiovascular disease, rheumatic diseases, COPD, obesity, and smoking (209–211). A hypercoagulable state with elevated pro-inflammatory and pro-coagulant factors, impaired endogenous fibrinolytic system, and platelet hyperactivity is also related to these stroke risk factors such as older age (212), smoking (213), obesity (214), hypertension (215), and diabetes (216). Small vessel disease due to hypertension, diabetes, and hyperlipidemia may disrupt the BBB (217). In addition, comorbidities such as diabetes, hypertension, and hyperlipidemia are risk factors for hypomagnesemia (218, 219) which in turn elevates the risk of cardiovascular disease and stroke (203, 220–222).

Liver has an essential role in hemostasis by producing the coagulation and anti-coagulation factors, and the regulation of platelet synthesis by producing thrombopoietin (223, 224). Chronic liver disease can lead to coagulation imbalance and both thrombotic and bleeding disorders (223, 224).

Patients with chronic kidney disease (CKD) are at elevated risk for stroke, both hemorrhagic and ischemic, and poor outcome after stroke (225). Accelerated atherosclerosis, endothelial dysfunction, impaired cerebral autoregulation, anemia, uremic toxins, hyperhomocysteinemia, proteinuria, impaired calcium/phosphate metabolism have been suggested to increase the risk of stroke in patients with CKD (225–227). Cerebral small vessel disease that may be a marker of a multi-system endothelial disorder has been associated with the severity of renal impairment (228). CKD was suggested to have a predictive role in the presence and severity of cerebral small vessel disease (229).

Patients with different types of malignancy are vulnerable to both hemorrhagic and ischemic stroke (230). Stroke may result from direct effects of a primary or metastatic tumor in the CNS, or indirect effects of chemotherapeutic agents, higher susceptibility to infection, and cancer-associated coagulopathy (230, 231).

Patients with neurotrauma or pre-existing neurologic conditions such as multiple sclerosis and Alzheimer's disease may have BBB breakdown lasting for a longer time potentially increasing the risk for viral neuroinvasion and neuroinflammation (217, 232, 233).

Thyroid hormones influence the cardiovascular system and thyroid imbalance is associated with vascular diseases including stroke (234, 235). Supraventricular dysrhythmias including AF are associated with both overt and subclinical hyperthyroidism with elevated risk for cardioembolism (234). Hypothyroidism has a depressive effect on the cardiac conduction system that may be beneficial in patients who are at risk for cardiac ischemia and arrhythmia (236). However, hypothyroidism causes autonomic dysfunction with adverse cardiovascular effects (237) and increases the risk for QT prolongation and atrioventricular block (236). Hypothyroidism, even at subclinical levels, has been associated with vascular risk factors such as hypertension and hyperlipidemia that promote atherosclerosis (234, 238, 239). A hypercoagulable state, although has not been established, may occur in hyperthyroidism with elevated levels of fibrinogen and von Willebrand antigen (234, 239). Through various mechanisms, either elevation or reduction in thyroid hormone levels causes endothelial dysfunction, hypertension, impaired cardiac function (239). The significant prevalence of subclinical thyroid disease, 3–12% for subclinical hypothyroidism and 1–6% for subclinical hyperthyroidism (240), suggests assessing the thyroid function in patients with features of severe COVID-19.

### Genetic Predisposition to Cardiovascular Disease and Stroke

Although SARS-CoV-2 infection makes patients with existing cardiometabolic diseases more vulnerable to have severe vascular consequences such as stroke, currently, there is no report of genome-wide association studies findings on stroke (ischemic stroke or hemorrhagic stroke) in COVID-19 patients. Whether individual genetic risk and polygenic theory for chronic cardiometabolic diseases and stroke play a role in the susceptibility to severe clinical manifestation in COVID-19 is still unknown.

The performance of polygenic risk scores (PRS) over the life course in several cardiometabolic diseases and neoplasms have been evaluated in a prospective setting and their value, when integrated with the known clinical risk factors and biomarkers, have been revealed (241). The cumulative risk for coronary artery disease, diabetes mellitus type 2, and AF was disproportionally increased after 40 years old when patients were stratified by categorical PRS from higher (>97.5%) to lower (<2.5%) scores. Genetic overlaps between stroke risk, early neurological changes, and some of the cardiovascular risk factors (diabetes and hypertension) have been identified (242). Because of the pleiotropy of genetic risk factors for both ischemic stroke and chronic diseases (243), we expect ischemic stroke will show a similar pattern. The cumulative disease rate for ischemic stroke will be disproportionally higher in the top PRS category. The polygenic contribution to early-onset was much higher than to late-onset in the same disease (241). Our retrospective study from the sensitivity analysis alternatively confirmed this disproportional increased PRS burden for ischemic stroke using younger cases vs. three tiers of older controls (from 59, 69 to 79) (243). Gene sets analyses highlighted the association of PRS with Gene Ontology terms (vascular endothelial growth factor, amyloid precursor protein, and atherosclerosis). All these pathways, as we reviewed here, could be potential targets of COVID-19. Future studies on this topic would help to lineate the potential capability of PRS in determining the genetic liability to the stroke or its subtypes as well as predicting the outcome in patients with COVID-19 infection. Whether patients having high PRS value for specific pathways may indicate the potential causal mechanism of ischemic stroke is still unknown and requires further investigation to validate. It is unclear to what extent PRS contributes to ischemic stroke in younger vs. older patient with COVID-19 infection.

### Congenital Predisposition to Cardiovascular Disease and Stroke

Inherited stroke syndromes are among the disorders affecting the circulation and predisposing the patients to stroke. Cerebral autosomal-dominant arteriopathy with subcortical infarcts and leukoencephalopathy (CADASIL) is the most common hereditary stroke disorder. A patient with COVID-19 and CADASIL presented with multiple acute small vessel infarctions in subcortical areas that highlight the vulnerability of high-risk patients for stroke during COVID-19 infection (244). Inherited thrombophilias such as factor V Leiden and protein C deficiency increase vascular thrombosis risk (245). Also, patients with inherited arrhythmia syndrome such as congenital long QT syndrome, and Brugada syndrome might be at greater risk for COVID-19-related arrhythmia (246). In addition, the prevalence of patent foramen ovale is reported in about 25% of the general population and twice more in people with cryptogenic stroke (247). Patent foramen ovale associated stroke represents an important consideration when taking into account the high incidence of venous thrombosis among COVID-19 patients in the ICU ranging between 40% (248), and 69% (249).

### Miscellaneous Risk Factors

Stress has been shown by a meta-analysis to be associated with a higher risk of cardiovascular mortality and stroke (250). This association is multifactorial and different neurohormonal mechanisms may be implicated. One mechanism might be the induction of toxic neuropeptides such as beta-amyloid that persists for a long time, activate the hypothalamic-pituitary-adrenal axis to produce a corticotropin-releasing hormone, increase neuroinflammation by activating mast cells (233, 250). Mast cells produce and respond to corticotropin-releasing hormone and can play a significant role in the cerebral pathologies in COVID-19 such as vascular dysfunction and BBB disruption (233). The incidence of stress cardiomyopathy (also known as Takotsubo syndrome) has been raised during the COVID-19 pandemic compared with the pre-pandemic period (251).

Cough is one of the most common presenting symptoms in COVID-19 (252). Though extremely rare, severe cough can suddenly increase intrathoracic pressure and cause a vascular rupture. A case of spontaneous spinal subarachnoid hemorrhage due to a severe cough that presented with a severe and sudden onset back pain and a headache was reported previously (253). A case of carotid artery dissection in a previously healthy adult patient was also reported during a course of severe cough due to pertussis (254).

Prosthetic valves and vascular prosthetic grafts are prone to thrombosis and subsequent vascular events. A patient with COVID-19 developed acute limb ischemia due to complete occlusion of the abdominal aortic prosthetic graft (255).

## Medications

Figure 6 demonstrates the medications that have been associated with either ischemic or hemorrhagic stroke. Here, we briefly cover these medications by considering the presence (section Medications with Potential Benefit for COVID-19 Therapy) or absence (section Medications without Possible Effects on COVID-19) of potential benefit on COVID-19 therapy.

### Medications With Potential Benefit for COVID-19 Therapy

Except for remdesivir, and a combination of baricitinib and remdesivir that have been recently approved for COVID-19 treatment (256, 257), the efficacy of other therapeutic agents in SARS-CoV-2 infected patients has not been approved. However, several medications are under investigation or being used off-label since the beginning of the pandemic (258). Some of these medications may be associated with serious complications such as stroke. Prolonged QT interval increases the risk of stroke independent of traditional risk factors (259). Administration of chloroquine and hydroxychloroquine (e.g., NCT04353336), favipiravir (e.g., NCT04359615, NCT04464408), lopinavir/ritonavir (e.g., NCT04372628, NCT04499677), atazanavir (e.g., NCT04468087, NCT04452565), remdesivir, and azithromycin (e.g., NCT04334382, NCT04359316), which are proposed as therapeutic options in SARS-CoV-2 infection, have the potential for QT interval prolongation and increasing the risk of arrhythmia (260–262). Some other medications from anesthetics, antipsychotics, antiemetics, and antiarrhythmics also have the QT prolongation potential. This complication is amplified by metabolism reduction (drug-induced, liver- and/or renal-induced), concomitant use of other medications with a potential for QT prolongation, electrolyte disturbance, and congenital long QT syndromes (263, 264).

Numerous drugs have the potential to inhibit the cytochrome P450 (CYP) system and increase the adverse effects of drugs that are inactivated by CYP enzymes. Azithromycin and antiviral agents such as lopinavir/ritonavir, atazanavir, remdesivir are inhibitors of CYP3A4 isoenzyme. The risk of QT prolongation is expected to be higher in combination use with other drugs that are metabolized by CYP3A4 such as chloroquine and hydroxychloroquine (265).

Antiretroviral protease inhibitors including lopinavir/ritonavir and atazanavir reduce viral replication and pro-inflammatory cytokines (266). Protease inhibitors are potent inhibitors of CYP, particularly CYP3A4, CYP2C9, and CYP2C19 (267). Protein inhibitors decrease the active metabolites of warfarin and increase the required warfarin maintenance dose (267), and change the active metabolites of antiplatelets (265, 268). For example, the antiplatelet function of clopidogrel was inadequate when administered in combination with antiretroviral agents (ritonavir and cobicistat, and atazanavir) (265, 268). Prasugrel remained effective despite a slight reduction in active metabolites (268). Thereby, when an antiplatelet is needed in combination with ritonavir, prasugrel is the best candidate. Conversely, ticagrelor is an activated medication and combined use with CYP inhibitors (such as ritonavir) increases its antiplatelet activity by 4-fold, which may induce bleeding (269). Combination therapy of ticagrelor with atazanavir is also restricted, atazanavir may increase the active metabolite of ticagrelor (265). The drug-drug interaction also exists between lopinavir/ritonavir and atazanavir, with direct oral anticoagulants such as rivaroxaban and apixaban (265). Patients infected with SARS-CoV-2 who were on direct oral anticoagulants showed a marked elevation in the plasma levels of anticoagulants after initiation of antiviral agents (270). Several antiarrhythmic drugs, such as amiodarone, lidocaine, and beta-blockers, are also metabolized by the hepatic CYP system. Close monitoring with measurement of serum drug concentration and electrocardiogram are recommended in a combinational regimen with antivirals (271).

Tumor necrosis factor alpha (TNF-α) inhibitors were proposed for the treatment of severe COVID-19 (272). From this family, Infliximab is being investigated for moderately or severely ill patients with COVID-19 (NCT04593940, NCT04425538). TNF-α inhibitors have been associated with the induction of antiphospholipid antibody syndrome and vasculitis (273, 274). These drugs are generally well-tolerated and the mentioned side effects are rare and reversible with drug cessation (274). However, arterial and venous thromboembolism have been reported in association with consumption of adalimumab, infliximab, and etanercept (275, 276). The observed thrombotic events with TNF-α inhibitors have been mainly related to the induction of antiphospholipid antibodies (277–279).

Tocilizumab is a monoclonal antibody that inhibits IL-6 receptors and is suggested as a therapeutic option for cytokine storm in COVID-19 pneumonia due to the prominent role of IL-6 in severe infection (280, 281) and is currently being investigated by several clinical trials (e.g., NCT04356937, NCT04445272). Although it may be beneficial in reducing the severity of stroke (282), there are rare cases of thrombotic microangiopathy involving multifocal cerebral lesions developed after administration of tocilizumab (283, 284). Tocilizumab was hypothesized as a trigger rather than a cause since patients had other risk factors for vascular events such as factor rheumatoid arthritis and factor V Leiden mutation.

Glucocorticoids are being used in the treatment of patients with severe COVID-19 (e.g., NCT04438980, NCT04395105, NCT04513184), whereas their use is associated with cardiovascular, cerebrovascular disease (285), and thromboembolic events with increased risk for AF (286).

Efficacy of high dose intravenous immunoglobulin (IVIG) has been observed in severe COVID-19 and has been suggested as a therapeutic option (287). IVIG is under evaluation by several clinical trials (e.g., NCT04432324, NCT04521309, NCT04480424). Previous evidence showed arterial and venous thrombosis after treatment with IVIG (288–291) even in young patients without risk factors for vascular diseases (290). Prothrombotic state in high dose IVIG therapy was attributed to hyperviscosity, vasospasm, platelet activation, and the presence of antiphospholipid antibodies and activated factor XI in some IVIG preparations (288–291).

Sildenafil, a cyclic guanosine monophosphate-specific phosphodiesterase type 5 inhibitor, is another candidate for the treatment of COVID-19 due to its role in inflammation reduction (NCT04489446, NCT04304313). Sildenafil was associated with a transient ischemic attack, ischemic stroke, and cerebral venous sinus thrombosis (292, 293). Endothelial dysfunction, hypoperfusion due to arterial dilation, and blood stasis due to vasodilation in patients with predisposing factors have been associated with vascular events in sildenafil users (292, 293). Rare cases of AF in patients with concomitant cardiac pathology have been related to sildenafil (294, 295).

Cannabis is used for its therapeutic effects in reducing chronic pain, nausea, and vomiting in patients under chemotherapy (296). Furthermore, it showed a beneficial role in animal models of ARDS by the reduction in pro-inflammatory cytokines, promotion of apoptosis in activated immune cells (297, 298), and upregulation of apelin (299), a peptide with protective role during ARDS (300). Due to the immunomodulatory effects of cannabis components, several clinical trials are registered to investigate its efficacy in the prevention and treatment of COVID-19 in addition to COVID-induced psychologic disorders such as anxiety and depression (NCT04467918, NCT03944447, NCT04731116, NCT04603781). On the other hand, several cases have been reported on the relationship between cannabis use and stroke and some of the cases were young and low risk for stroke (301, 302). However, the observed association may be related to concomitant smoking or the use of other unknown toxins in synthetic marijuana (302, 303). Cannabis exposure may be related to cerebral vasoconstriction, cardioembolism due to cardiac ischemia and arrhythmia, and thrombophilia (304, 305). Cannabis may rarely cause hemorrhagic stroke due to a sudden rise in blood pressure (305).

Heparin is the most widely used anticoagulant which can also be classified as an anti-inflammatory agent due to the inactivation of pro-inflammatory cytokines (306). In addition, the interaction of heparin with SARS-CoV-2 S1 spike protein supports the repurposing of heparin as an antiviral agent (307). Therefore, heparin has been added to the list of medications under investigation for COVID-19 (eg, NCT04530578, NCT04485429). Although heparin has a satisfactory safety profile and the risk of major bleeding events is low, there are some rare reports on severe and fatal bleeding such as intracranial hemorrhage in association with heparin administration (308, 309). Heparin-induced thrombocytopenia (HIT) is a relatively rare immune-mediated adverse reaction of heparin indicating by low platelet counts, as a result of anti-heparin-platelet factor 4 (PF4) antibodies, and arterial and venous thrombosis in almost 50–70% of patients with HIT (310). Thrombocytopenia and hypercoagulability are shared features betweenCOVID-19 coagulopathy and HIT. HIT is a life-threatening etiology for thrombocytopenia and hypercoagulability that appears to have a higher incidence during COVID-19. Among critically ill patients with COVID-19, the incidence of HIT was reported to be about 8%, which was 10-fold higher than control patients without COVID-19 (311). False-positive results of HIT during COVID-19 was also suggested by a study due to reasons such as the high prevalence of antiphospholipid syndrome and inaccurate laboratory assessment (312). There is a report of five patients with positive anti-heparin PF4 antibodies but confirmatory test for HIT returned positive for only one patient. The authors attributed the observation to the production of anti-heparin PF4 antibodies as a consequence of severe COVID-19 which was suggested by another study (313). To avoid overdiagnosis of HIT and unnecessary discontinuation of heparin, they recommended using the standard tests instead of antibody assessment alone (312).

Tissue plasminogen activator (t-PA) is under investigation for treating ARDS in COVID-19 (e.g., NCT04356833, NCT04357730). t-PA is the standard treatment for acute ischemic stroke that may adversely cause intracranial hemorrhage (314). Surprisingly, t-PA can cause a secondary hypercoagulability and increase the risk for thrombotic events (315).

Ivermectin is an anthelmintic agent with *in vitro* inhibition of SARS-CoV-2 replication (316). It is proposed as a treatment for COVID-19 and is under investigation (e.g., NCT04529525, NCT04425707). This medication was attributed to coagulopathy, possibly through interaction with coagulation factors (e.g., factors II and VII). This adverse reaction has been related to a case of warfarin toxicity after treatment with ivermectin (317).

Vascular endothelial growth factor (VEGF) antagonists are related to thrombotic and hemorrhagic side effects (318). VEGF-induced angiogenesis plays a significant role in acute lung injury of COVID-19 (319). For this reason, bevacizumab -an anti-VEGF agent- is under examination for the inhibition of angiogenesis in COVID-19 pneumonia (NCT04275414, NCT04344782, NCT04305106). Bevacizumab has been previously associated with venous and arterial thrombosis and hemorrhage, including stroke (320, 321).

### Medications Without Possible Effects on COVID-19

In addition to medications under trials for SARS-CoV-2 infection, there are other medications with elevated risk for stroke. Prior routine consumption of these medications or use for acute conditions unrelated to COVID-19, such as trauma or surgery, might increase the risk of stroke which in addition to other stroke risk factors can lead to an attack in a patient with SARS-CoV-2 infection. Some of these medications, which are more widely used, are discussed here to remind the clinicians of other drugs with elevated risk of stroke particularly cryptogenic stroke.

AF can be stimulated by several mechanisms including medications. As discussed in earlier sections, patients with COVID-19 are prone to cardiovascular damage and new-onset arrhythmia as a consequence of comorbidities and infection. Drugs that have been associated with the induction of AF and the possible mechanisms are comprehensively reviewed by previous studies (322–324). Based on these studies, drug-induced AF is believed to have the following principal mechanisms: alterations in autonomic tone through either adrenergic or vagal stimulation, changing the atrial automaticity and conduction, direct cardiovascular toxicity such as coronary vasoconstriction/ischemia and electrolyte disturbances, local or systemic inflammation, oxidative stress, hyperthermia (322–324). A comprehensive discussion of all medications that are being associated with AF in addition to the possible mechanisms are not in the scope of this review; however, more commonly used medications are presented in Figure 6.

Some chemotherapeutic agents such as busulfan, bleomycin, cis-platinum, and fluorouracil increase the risk of thrombosis (325) and are also associated with cardiotoxicity (326). Angiogenesis inhibitors are also reported to be cardiotoxic with elevated risk for hypertension, arterial thromboembolism, and myocardial ischemia (327). L-asparaginase is an antineoplastic agent that causes a hypercoagulable state leading to thrombotic events. This drug causes reduced protein synthesis such as antithrombin III (328).

Exogenous estrogens, oral contraceptives, hormone replacement therapy, and estradiol produced from the conversion of exogenous testosterone are associated with the risk of venous and arterial thrombosis mainly through induction of hypercoagulability (329, 330). Tamoxifen has also an estrogenic effect and may be related to both venous thromboembolism and stroke (331).

Supraphysiologic doses of anabolic steroids are reported to cause ischemic stroke and myocardial infarction mostly in young patients with unremarkable vascular risk factors (332–334). A thrombogenic and an atherogenic state have been proposed as a result of AF, increased erythropoietin and hematocrit, increased thrombin and fibrin formation and platelet aggregation, vascular spasm, hypertension, insulin resistance, and dyslipidemia (332, 335, 336).

Antipsychotics are associated with increased risk for both stroke and MI possibly related to platelet aggregation, metabolic effects, and obesity (337). Serotonin is a vasoactive amine with both vasoconstrictive and vasodilative effects on cerebral arteries of different sizes (325). Selective serotonin reuptake inhibitor (SSRI) antidepressants and new antipsychotics such as clozapine and olanzapine have been reported to cause cardiodepression and arrhythmia (338). Based on a meta-analysis, patients with a one-time stroke under antidepressant therapy (including SSRI and tricyclic antidepressants) may be at elevated risk for stroke recurrence, particularly ischemic type. The risk was highest in patients under therapy with multiple antidepressants (339). SSRI use has also been shown to cause an elevated risk of ischemic stroke in patients 65 years and older (340). Platelets release serotonin at the site of endothelial injury to promote platelet aggregation (341). SSRI may decrease serotonin reuptake by platelets and cause platelet dysfunction and bleeding. Several studies have supported the increased risk of bleeding in patients with SSRI use (342, 343). According to a meta-analysis, SSRI is associated with a slightly increased risk of hemorrhagic cerebral events which is potentiated with concomitant use with oral anticoagulants (344).

Opioids, although rarely reported, are accused of being associated with ischemic stroke. Possible mechanisms might be cardioembolism due to recurrent infective endocarditis, hypotension, and bradycardia leading to global hypoperfusion and ischemia, and vasculitis (345).

Some of the illicit drugs including amphetamines and cocaine cause elevated incidence and mortality of stroke by sympathomimetic activity (346). Vascular spasm, an acute rise in blood pressure, aneurysm formation and rupture, and vasculitis are the reasons by which sympathomimetics may stimulate a stroke (346). Chronic use of these illegal sympathomimetics stimulates ischemic stroke. The proposed mechanisms are vasospasm and tissue hypoperfusion, accelerated atherosclerosis, increased platelet aggregation, vasculitis and cardioembolism due to infective endocarditis, arrhythmia, cardiomyopathy and myocardial infarction (305, 347). Sympathomimetic compounds in over-the-counter cold medications and appetite suppressants such as phenylpropanolamine and pseudoephedrine are also increase the risk of hemorrhagic and ischemic stroke and myocardial infarction (348, 349). Vascular events are more probable in chronic drug consumption or in higher doses than the recommended dose and the proposed mechanisms are related to sympathomimetic effects such as vasospasm and hypertension (348, 349).

## Discussion and Future Perspective

Patients with SARS-CoV-2 infection need prevention for vascular events, especially during hospitalization. According to the International Society of Thrombosis and Hemostasis, all hospitalized patients infected with SARS-CoV-2 benefit from coagulation monitoring with D-dimer level, prothrombin time, platelet count, and fibrinogen. Prophylactic low molecular weight heparin therapy is also beneficial (350). Considering the hypercoagulability in COVID-19, higher doses of anticoagulants with extended duration of anticoagulation may be needed, and laboratory and clinical follow-ups should occur in shorter intervals. Notably, a high incidence of arterial and venous thrombosis occurs despite thromboprophylaxis (89, 249). Among a cohort of infected patients who were discharged without anticoagulants, 2.5% presented with a thrombotic event post-discharge (mean of 30 days), including one ischemic stroke at day 40 post-discharge (351). However, therapeutic or prophylactic doses of anticoagulation may result in severe and fatal intracranial hemorrhage in SARS-CoV-2 infected patients (89, 352), possibly due to endotheliopathy and vascular fragility. Based on the current literature, it cannot be anticipated for how long COVID-19 survivors would be prone to the elevated risk of stroke. Previous experiences with other pathologies may provide an insight into the duration of the preventive strategies. Follow up of hospitalized pneumonia patients revealed an increased risk of cerebro- and cardiovascular diseases, even up to 10 years. After adjustment for cardiovascular risk factors, the risk was highest in the first month, had been progressively declined in the first year, and remained as high as 1.5-fold of the risk in the controls in the following years (353). Similarly, hospitalization for sepsis increased the 1-year risk of stroke, particularly in younger patients (354). In the influenza-like illnesses, the higher odds of ischemic stroke was significant up to 60 days (355). Study on twenty-five SARS-CoV-1 survivors after 12 years showed a significant disruption in the lipid metabolism, cardiovascular abnormalities, and altered glucose metabolism, compared to healthy controls (356).

Finally, the role of antiplatelet agents in the secondary prevention of stroke is clear (357). Patients with SARS-CoV-2 infection may further benefit from antiplatelets due to the role of platelets in the coagulopathy related to severe COVID-19. On the other hand, platelets are essential components of defense against viruses and antiplatelets may inhibit the immune system in the early stages of viral infection complicating antiplatelet therapy. In this respect, previous studies have shown the lack of cytotoxic T cell-mediated response and inhibition of viral clearance in platelet-depleted animals (358) and also in animals treated with dual aspirin/clopidogrel (359). Due to the lack of clear evidence, it is most reasonable to personalize patient care. While waiting for the results of standard trials for COVID-19 treatment, uncontrolled administration of several drugs may increase the risk for adverse reactions such as stroke.

In summary, the present narrative review highlighted the potential pathways that may be associated with CNS invasion by SARS-CoV-2. We pictured the potential etiopathogenic mechanisms that may account for the causative association between COVID-19 and stroke. Further research is required to assist us in understanding the full spectrum of SARS-CoV-2 infection in triggering acute cardio- and cerebrovascular events.

## Author Contributions

SASN, SS, VA, and RZ contributed to the conception and design of the study. SASN, SS, EK, IF, FKh, MS, FKo, and SA performed the literature review, wrote the first draft of the manuscript, and prepared the figures. GT, JL, VA, DW, and RZ wrote sections of the manuscript and performed the initial revision. All authors contributed to the final revision of the manuscript and figures and read and approved the submitted version.

## Conflict of Interest

The authors declare that the research was conducted in the absence of any commercial or financial relationships that could be construed as a potential conflict of interest.
